# Machine Learning for Head and Neck Cancer: A Safe Bet?—A Clinically Oriented Systematic Review for the Radiation Oncologist 

**DOI:** 10.3389/fonc.2021.772663

**Published:** 2021-11-18

**Authors:** Stefania Volpe, Matteo Pepa, Mattia Zaffaroni, Federica Bellerba, Riccardo Santamaria, Giulia Marvaso, Lars Johannes Isaksson, Sara Gandini, Anna Starzyńska, Maria Cristina Leonardi, Roberto Orecchia, Daniela Alterio, Barbara Alicja Jereczek-Fossa

**Affiliations:** ^1^ Division of Radiation Oncology, European Institute of Oncology (IEO) Istituto di Ricovero e Cura a Carattere Scientifico (IRCCS), Milan, Italy; ^2^ Department of Oncology and Hemato-Oncology, University of Milan, Milan, Italy; ^3^ Molecular and Pharmaco-Epidemiology Unit, Department of Experimental Oncology, European Institute of Oncology (IEO) Istituto di Ricovero e Cura a Carattere Scientifico (IRCCS), Milan, Italy; ^4^ Department of Oral Surgery, Medical University of Gdańsk, Gdańsk, Poland; ^5^ Scientific Directorate, European Institute of Oncology (IEO) Istituto di Ricovero e Cura a Carattere Scientifico (IRCCS), Milan, Italy

**Keywords:** systematic review, artificial intelligence, machine learning, radiotherapy, head and neck cancer

## Abstract

**Background and Purpose:**

Machine learning (ML) is emerging as a feasible approach to optimize patients’ care path in Radiation Oncology. Applications include autosegmentation, treatment planning optimization, and prediction of oncological and toxicity outcomes. The purpose of this clinically oriented systematic review is to illustrate the potential and limitations of the most commonly used ML models in solving everyday clinical issues in head and neck cancer (HNC) radiotherapy (RT).

**Materials and Methods:**

Electronic databases were screened up to May 2021. Studies dealing with ML and radiomics were considered eligible. The quality of the included studies was rated by an adapted version of the qualitative checklist originally developed by Luo et al. All statistical analyses were performed using R version 3.6.1.

**Results:**

Forty-eight studies (21 on autosegmentation, four on treatment planning, 12 on oncological outcome prediction, 10 on toxicity prediction, and one on determinants of postoperative RT) were included in the analysis. The most common imaging modality was computed tomography (CT) (40%) followed by magnetic resonance (MR) (10%). Quantitative image features were considered in nine studies (19%). No significant differences were identified in global and methodological scores when works were stratified per their task (i.e., autosegmentation).

**Discussion and Conclusion:**

The range of possible applications of ML in the field of HN Radiation Oncology is wide, albeit this area of research is relatively young. Overall, if not safe yet, ML is most probably a bet worth making.

## Introduction

Cancers of the head and neck (HN) region involve anatomically complex and functionally essential structures, whose damage may severely compromise quality of life, especially in long-surviving patients (1). If the management of HN cancers (HNCs) has always been challenging in Radiation Oncology, in the last years, the clinical scenario has rapidly evolved, due to changes in the epidemiology of the disease (2–4), to the introduction of novel systemic therapies and surgical procedures (5–8) and to the availability of more sophisticated irradiation techniques (9–11). Additionally, as for other cancer sites, understanding on HN neoplasms is taking advantage from progresses in the fields of radiogenomics and quantitative imaging analysis (12–15). Such “big data”-based approaches are progressively being integrated into a more traditional body of knowledge on tumor biology and inter-patient variability which, arguably, may represent a concrete step toward a personalized medicine approach (16).

Nevertheless, this increasing amount of information is hardly manageable by single practitioners, and there is an unprecedented demand of novel, informatics-based tools to structure and solve complex clinical questions. To this aim, machine learning (ML)—a branch of artificial intelligence (AI) relying on patterns and inference to execute a specific task—could provide Radiation Oncologists (ROs) with accurate models to optimize patients’ care paths (17).

As compared with statistical methods, ML focuses on the identification of predictive patterns rather than on drawing inferences from a sample. Starting from sampling and power calculations, statistical models aim to assess whether a relationship between two or more variables describes a true effect and to interpret the extent of the above-mentioned relationship. A quantitative measure of confidence can therefore be provided to test hypothesis and/or verify assumptions (18). By contrast, ML makes use of general-purpose algorithms with no or minimal assumptions. While this may produce hardly interpretable and generalizable results, ML can be useful in case of poorly understood and complex phenomena, when the number of input variable exceeds the number of subjects and complicated nonlinear interactions are present (19). However, statistics- and ML-based models should not be regarded as antagonistic and mutually exclusive. As an example, some methods (i.e., bootstrapping) can be used for both the purpose of statistical inference and for the development of ML models, and a distinct boundary between the two is not always easily traceable.

The choice of the most suitable ML algorithm to solve a given problem starts with the characterization of available data, which can be either labeled (e.g., implemented with additional information, such as: “this computed tomography (CT) slice contains the contour of the tumor”) or unlabeled (e.g., data do not contain any supplementary tag, such as a collection of CT slices). In the first case, the learning problem is of supervised nature, meaning that the algorithm uses labeled data (training set) to assign a class label to unseen, unlabeled instances (test set). Conversely, unsupervised learning uses unlabeled data to identify previously undetected patterns in the data set and reacts to the existence or absence of such patterns in new instances, without the need of human supervision. However, the aim of the model is the same: to assign similar, contiguous pixels with the correct label (PG vs. non-PG) by a computationally efficient and generalizable algorithm. Other than by input data type, models can be categorized according to their output. Broadly, if the output is a number (i.e., grade of acute toxicity per the Common Terminology Criteria of Adverse Events (CTCAE) system), the task is defined as a regression problem, if it is a class (i.e., tumor vs. nontumor), the task is called a classification problem, and if it is a set of input groups (i.e., clinical and dosimetric variables), it is a clustering problem.

**Figure 1:**
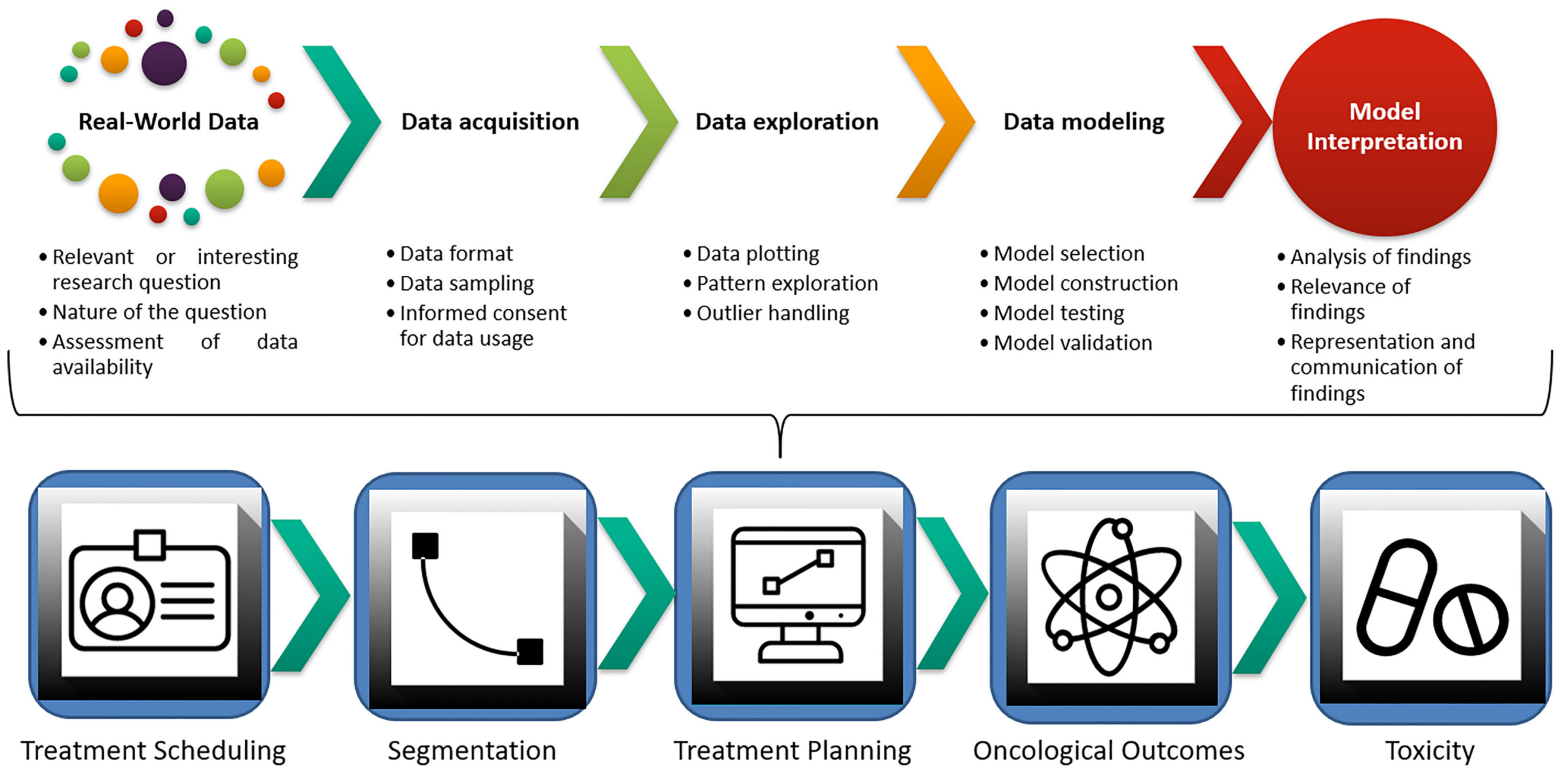
Machine learning workflow and current applications in Radiation Oncology.

Following the idea of a “big-data” approach for cancer care, several publications in the field of Radiation Oncology have come to life, with algorithms encompassing segmentation accuracy, treatment planning optimization, and prediction of both oncological and toxicity outcomes (17, 20–22). A visual representation of the ML workflow applied in this clinical setting is provided in **Figure 1**. Given the lack of comparable efforts in current literature and the hotness of the topic, we decided to perform a clinically oriented systematic review of the available evidence for ML applications in HNCs. In doing so, we also chose to focus on the methodology of published works and to rate their quality according to a ML-dedicated checklist by Luo et al. (23), generated in 2016 by a multidisciplinary panel of experts in compliance with the Delphi method (24). Ultimately, our goal is to propagate awareness of ROs on ML applications in HNCs. Expectantly, this would contribute to fostering further research and collaboration among different professionals, and to define a novel, data-driven approach to clinical Radiation Oncology for this subset of patients.

### Autosegmentation

Segmentation of target volumes and organs at risk (OARs) is a critical component in the Radiation Oncology workflow. Following the recognition of intensity-modulated radiotherapy (IMRT) as a standard of care for HNC (25), accurate delineation has been associated with improved oncological and toxicity outcomes (26–28). Consequently, minimizing inter- and intraoperator variability in segmentation is crucial, and several guidelines have been published and updated to foster standardization in HNC contouring. Another relevant issue in the current clinical management is the time needed for completing the segmentation of an HNC case, which approximates 3.0 h (29): other than representing a significant commitment to the RO, time represents a limitation toward a more systematic use of adaptive radiotherapy (ART), which requires rapid recontouring and replanning (30). In this context, ML-based autosegmentation holds the promise of optimizing the clinical management for HNC patients and to increase consistency and reproducibility of delineated structures. ML can be implemented to either single or multiple autosegmentation atlases in order to improve registration and segmentation performance. Specifically, such model-based approaches can compare patient’s images with a reference gold standard (ground truth) and overcome acquired imaging limitations including low soft tissue contrast and presence of dental metal artifacts. However, inter- and intrapatient variability and large computational time for registration represent two significant pitfalls of the atlas-based approach (31). Deep learning has the potential to overcome these limitations and has already found several applications in the field of computer vision tasks which, as a whole, can be defined as the automatic extraction, analysis, and understanding of any relevant information from either a single image or a series of images through the construction of dedicated datasets (21, 32).

### Treatment Planning

Treatment planning for HNC is challenging: expertise in both the medical (i.e., knowledge of complex HN anatomy and patterns of disease recurrence, awareness of tolerance of healthy tissues to irradiation) and in the physical field (i.e., coverage of irregularly shaped target volumes, multiple dose prescription levels) is required, and timely delivery of radiotherapy (RT) is mandatory not to compromise oncological outcomes (33). In recent years, an increasing body of evidence has demonstrated that geometrical and anatomical variations can occur during the course of curative-intent treatments for HNC, thus leading to potentially meaningful modifications in dose distribution. Several variables have been investigated, and include, but are not limited to, patients’ weight loss, tumor response, and PG shrinkage (34, 35). The use of ART can quantify and overcome the dosimetric impact of these modifications and restore the desirable therapeutic ratio in this subset of patients (36). Yet, routine implementation of ART in clinical practice is limited by temporal and logistic issues: CT rescanning, recontouring, and replanning require efficient scheduling and execution and involve the whole staff of a Radiation Oncology Department, from radiation therapists to medical physicists.

### Oncological Outcome Prediction

Outcome prediction is crucial in the field of Radiation Oncology, especially in the era of personalized treatments. As deintensification strategies are being tested in clinical trials (37), and biological and quantitative imaging parameters are gaining the spotlight as promising prognosticators (38, 39), there is an increasing need for effective models integrating this growing body of information (13). A typical problem in outcomes prediction with ML is the management of time-dependent endpoints (i.e., overall survival (OS), local control, progression-free survival). These outcomes, often referred to as “right censored”, may not have yet occurred at the time of the last follow-up, but still require to be considered, as they could present at a later time. Although the pre-processing method for such variables is often influenced by the ML algorithm of choice, it has been recognized that inappropriate recognition of right-censored events may lead to poorly calibrated models (40–43).

### Toxicity Outcomes Prediction

Other than achieving disease control by the irradiation of the gross and clinical tumor volumes (GTV and CTV, respectively), the optimal radiation treatment plan aims at the preservation of healthy surrounding structures. Although the introduction of modern RT techniques has ameliorated the therapeutic ratio, acute and chronic RT-related toxicities still represent a significant burden for patients’ quality of life and may compromise timely treatment delivery (25). In recent years, refined anatomical knowledge of normal tissues (i.e., the coexistence of serial and parallel components in architecturally complex patterns in salivary glands) and the recognition of a stem cell compartment in healthy organs have shed light on the need of further improving dose distribution, especially when curative-intent treatments are delivered (44).

To this aim, the use of spatial dose metrics, such as gradient and direction, may provide more comprehensive information than the sole absolute mean and maximum doses (45, 46). Additionally, genetic determinants are thought to impact on individual radiosensitivity/radioresistance of healthy tissues as much as for the 80% (47). ML may combine these emerging factors with more established determinants of toxicity, such as patient factors, administration of systemic therapies and absolute dosimetric parameters (48, 49). Adequate consideration of these covariables in dedicated algorithms could discriminate the probability for a given patient to experience a specific toxicity, and therefore contribute to refine clinical decisions (i.e., prophylactic feeding tube positioning in patients at high risk for severe weight loss) (47, 50).

## Materials and Methods

Study methodology complied with the outlines of the Preferred Reporting Items for Systematic Reviews and Meta-Analyses (PRISMA) (51). Original manuscripts on ML applications for HNC were considered eligible for the analysis; publications encompassing any other cancers were excluded. Interventions included investigations on (auto)segmentation, treatment planning, and outcome prediction (either oncological or toxicity); works whose focus was exclusively diagnostic were considered beyond the scope of the current review. Full papers of any study design except systematic reviews and case reports were considered; only works written in English were included.

### Search Strategy

Electronic databases (namely, National Center for Biotechnology Information PubMed, Elsevier EMBASE and Elsevier Scopus) were screened up to May 2021 without date restrictions by an author experienced in bibliographic search (SV). Free text, Boolean operators, truncation, and proximity operators were tested. No filters were applied, in order not to exclude potentially relevant publications. The full-search strategy is provided in **Supplementary Materials S1**.

**Figure 2:**
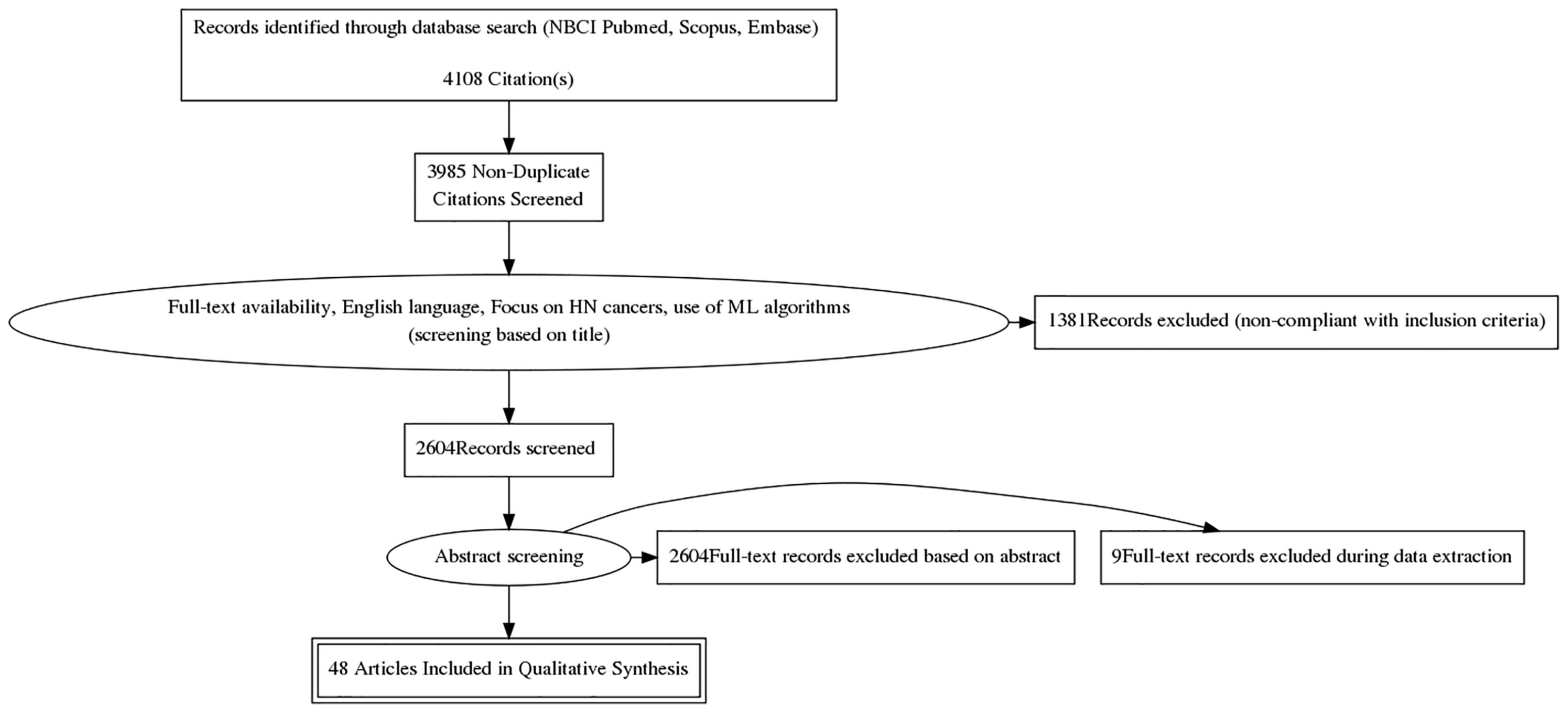
Study selection process per the Preferred Reporting Items for Systematic Reviews and Meta-Analysis (PRISMA) guidelines.

**Figure 3:**
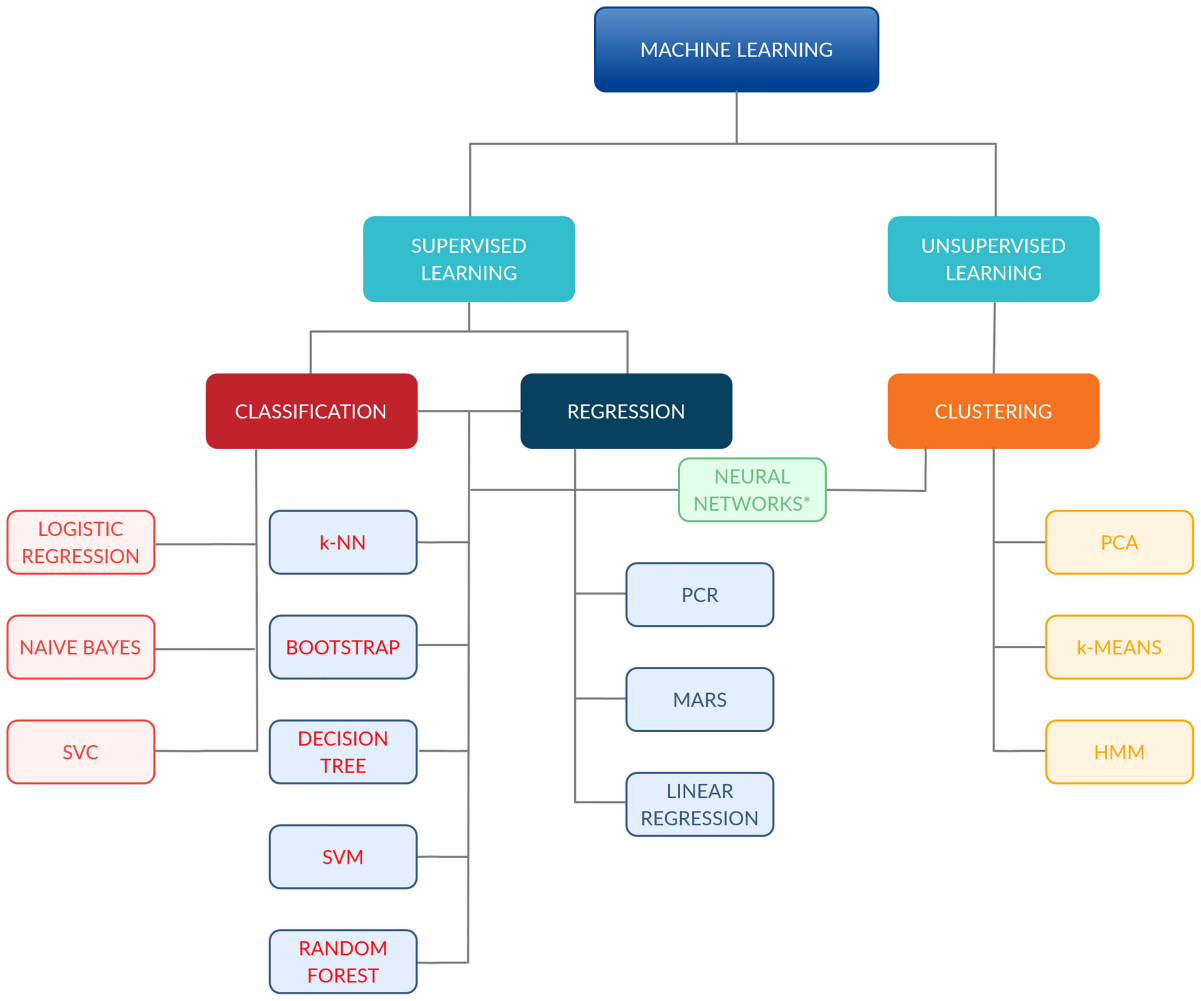
Classification of the machine-learning algorithms included in the analysis. ^*^Comprehend: ANN, CNN and FCNN. ANN, Artificial Neural Network; CNN, Convolutional Neural Network; FCNN, Fully CNN; HMM, Hidden Markov Model; k-NN, k-Nearest Neighbour; MARS, Multiadaptive Regression Splines; PCA, principal component analysis; PCR, principal component regression; SVC, support vector classifier; SVM, support vector machine.

Findings from the above-reported search were independently screened and selected based on titles by two Authors (SV, RS); disagreements were subsequently discussed in presence of three other authors (FB, MP, MZ). All types of ML algorithms were considered eligible for the analysis, as well as studies encompassing the use of extracted quantitative imaging features. The selection process is shown in **Figure 2**, while **Figure 3** provides an overview of the algorithms considered for the analysis. A more detailed insight of ML models/algorithms included is provided in **Table 1**.

**Table 1:** Summary and definitions of most common machine learning (ML) models.

ML model	Abbreviations	Application	Definition
**Artificial Neural Network**	ANN, NN	*Classification, regression, and clustering*	Any set of algorithms modeled on human brain neuronal connections
**Active Shape Model**	ASM	*Segmentation*	Model-based method to compare an image reference model with the image of interest
**Bayesian Bagging (Bootstrap AGGregatING)**	BB	*Classification and regression*	Bayesian analog of the original bootstrap. Bootstrap samples of the data are taken, the model is fit to each sample, and the predictions are averaged over all of the fitted models to get the bagged prediction
**Boosting**	–	*Classification and regression*	Boosting is a generic algorithm rather than a specific model. Boosting needs a weak model (e.g., regression, shallow decision trees, etc.) as a starting point and then improves it
**Bootstrap aggregating**	–	*Classification and regression*	Meta-algorithm designed to improve the stability and accuracy of ML algorithms used in statistical classification and regression. It also reduces variance and helps to avoid overfitting. Although it is usually applied to decision tree methods, it can be used with any type of method
**Classification and Regression Tree**	CART	*Classification and regression*	Predictive model which predicts an outcome variable value based on other values. A CART output is a decision tree where each fork is a split in a predictor variable and each end node contains a prediction for the outcome variable
**Convolutional Neural Network (CNN)**	CNN, NN	*Classification, regression, and clustering*	Ordinary NN which implements convolution (mathematical operation on 2 functions producing a third function expressing how the shape of the first one is modified by the second one), in at least 1 of its layers. Most commonly, inputs are images
**C4.5**	–	*Classification*	An algorithm used to generate a decision tree. The decision trees generated by C4.5 can be used for classification, and for this reason, this algorithm is often referred to as a statistical classifier
**Decision tree**	DT	*Classification and regression*	Algorithm containing conditional control statements organized in the form of a flowchart-like structure, also called tree-like model. Paths from roots to leaves represent classification rules, while each node is a class label (decision based on the computation of the attributes)
**Decision stump**	DS	*Classification and regression*	Model consisting of a 1-level decision tree, a tree with an internal node (root) immediately connected to the terminal nodes (its leaves). A DS makes a prediction based on the value of just a single input feature. Sometimes they are also called 1xrules
**Fully Convolutional Neural Network**	FCNN	*Classification, regression, and clustering*	A deep learning model based on traditional CNN model. A FCNN is one where all the learnable layers are convolutional, so it does not have any fully connected layer.
**Incremental Association Markov Blanket**	IAMB	*Features selection*	Feature selection method
**Least Absolute Shrinkage and Selection Operator**	LASSO	*Feature selection*	A regression analysis method that performs both variable selection and regularization in order to enhance the prediction accuracy and interpretability of the statistical model
**Likelihood-Fuzzy Analysis**	LFA	*Classification*	A method used for translating statistical information coming from labeled data into a fuzzy classification system with good confidence measure in terms of class probabilities and interpretability of the fuzzy classification model, by means of semantically interpretable fuzzy partitions and if–then rule
**Linear discriminant analysis**	LDA	*Classification*	A method used to find a linear combination of features that characterizes or separates 2 or more classes of objects or events
**Logistic regression**	LR	*Classification*	A statistical model that uses a logistic function to model a binary dependent variable
**k-Nearest Neighbors**	k-NN	*Classification and regression*	Non-parametric algorithm that classifies data points based on their similarity (also called distance or proximity) with the objects (feature vectors) contained in the collection of known objects (vector space or feature space)
**Multiadaptive Regression Splines**	MARS	*Regression*	It is a nonparametric regression technique, extension of linear models that automatically models nonlinearities and interactions between variables
**Multivariate Regression Model for Reserving**	MRMR	*Features selection*	Supervised feature selection algorithm which requires both the input features, and the output class labels of data. Using the input features and output class labels, MRMR attempts to find the set of features which associate best with the output class labels, while minimizing the redundancy between the selected features
**Naive Bayes**	NB	*Classification*	Applies Bayes’ theorem to calculate the probability of an hypothesis to be true assuming prior knowledge and a strong (therefore, naive) degree of independence between the features
**Partial least squares and principal component regression**	PLSR and PCR	*Regression*	Both methods model a response variable when there are a large number of predictor variables, and those predictors are highly correlated. Both methods construct new predictor variables, known as components, as linear combinations of the original predictor variables. PCR creates components to explain the observed variability in the predictor variables, without considering the response variable at all. PLSR does take the response variable into account, and therefore often leads to models that are able to fit the response variable with fewer components
**Principal component analysis**	PCA	*Clustering*	Captures the maximum variance in the data into a new coordinate system whose axes are called “principal components,” to reduce data dimensionality, favor their exploration, and reduce computational cost
**Penalized logistic regression**	PLR	*Classification*	PLR imposes a penalty to the logistic model for having too many variables. This results in shrinking the coefficients of the less contributive variables toward zero. This is also known as regularization
**Random forest (RF)/Random forest classification (RFC)**	RF, RFC	*Classification and regression*	Operates by constructing a multitude of decision trees at training time and outputting the class that is the mode of the classes (classification) or mean prediction (regression) of the individual trees
**Relief**	–	*Features selection*	An algorithm that takes a filter-method approach to feature selection that is notably sensitive to feature interactions. Relief calculates a feature score for each feature which can then be applied to rank and select top scoring features for feature selection
**Random survival forest**	RSF	*Survival*	A nonparametric method for ensemble estimation constructed by bagging of classification trees for survival data, has been proposed as an alternative method for better survival prediction and variable selection
**Rescorla Wagner model**	RW	*Classification, clustering*	Rescorla Wagner model is a model of classical conditioning, in which learning is conceptualized in terms of associations between conditioned and unconditioned stimuli
**Stochastic/Gradient Boosting**	–	*Classification and regression*	A ML technique which produces a prediction model in the form of an ensemble of weak prediction models, typically decision trees
**Support Vector Classifier**	SVC	*Classification*	The objective linear SVC is to fit to the provided data and returns a “best-fit” hyperplane that divides, or categorizes them
**Support vector machine**	SVM	*Classification and regression*	The SVM is based on the idea of finding a hyperplane that best divides the support vectors into classes. The SVM algorithm achieves maximum performance in binary classification problems, even if it is used for multiclass classification problems
**U-net architecture**	–	*Segmentation*	U-Net is a CNN that was developed for biomedical image segmentation. The main idea is to supplement a usual contracting network by successive layers, where pooling operations are replaced by up sampling operators. Hence, these layers increase the resolution of the output. A successive convolutional layer can then learn to assemble a precise output based on this information

### Quality Assessment of the Included Studies

The quality of the studies included in the analysis was rated by an adapted version of the qualitative checklist originally developed by Luo et al. for the reporting of predictive modeling in biomedical research (23). This checklist, compared with others present in the literature, provides a multidisciplinary overview of ML models, as it was developed taking into account inputs from different professional figures usually involved in medical research, such as clinicians, statisticians, and ML experts. The organization of the checklist was maintained, and the following subsections were rated for each study: “Title and abstract”, “Introduction”, “Methods”, “Results”, and “Discussion”. Each of the 55 items required a dichotomous answer (yes or no, coded as 1 and 0, respectively); two items were divided into three subsections, thus allowing for a maximum achievable score of 58. The complete adapted Luo scoring system can be reviewed in detail in **Supplementary Materials S2**.

### Statistical Analysis

Descriptive statistics (median, mean, interquartile range (IQR), min, max, standard deviation) were provided for global score and methodological score from the modified Luo classification (23). Score differences across study groups (per task and use of quantitative imaging analysis) were assessed with Wilcoxon sum-rank test (when groups = 2) or Kruskal-Wallis test (when groups >2) and graphically evaluated with boxplots. *p*-values corrected for false-discovery rate (FDR) were also provided to account for multiple testing, considering a threshold of 0.05. All statistical analyses were carried out using R version 3.6.1.

## Results

Forty-eight studies were included in the analysis: publication years ranged between 1998 and 2021; with more than a half having been published after 2018 (56%). Twenty-one (44%) focused on ML algorithms for autosegmentation, four (8%) were dedicated to treatment planning, 12 (25%) to oncological outcomes prediction, 10 (21%) to RT-related toxicity, and one (2%) to the determinants of postoperative RT delays following surgery for HNC.

Twenty-one works (44%) considered more than one HNC subsite, while the most common single primary site was the nasopharynx, which was the focus of seven studies (15%). Of note, this information was missing in six cases (12%). The most common imaging modality was CT (40%), followed by magnetic resonance (MR) (10%). Quantitative image features were considered in nine studies (19%) and were mainly CT based (75%). Dosimetric parameters were used in six of the analyzed works, five on toxicity outcomes prediction, and one on the identification of candidates to replanning.

Here follows a detailed description of the studies sorted by main topic, with each topic representing a critical step in the modern workflow for HNC patients in Radiation Oncology.

### Autosegmentation

The majority of the included studies (21/48) focused on the design of ML algorithms for autosegmentation: seven were for the segmentation of treating volumes (either CTV or GTV) and 13 for OARs. Considering the former, tumor GTV (GTV-T) was the target of prediction for six studies; in one of these, the algorithm was used for the delineation of the nodal GTV (GTV-N) and the CTV as well. Additionally, one study aimed at the sole segmentation of the left and right II–IV nodal levels. A fully automated approach was used in all but one study (52). Overall, all models included in the analysis compared favorably with either competing, previously published algorithms, or with the ground truth represented by manual segmentation (52–55). Specifically, the latter showed an overlap with the manual contours measured by the Dice Similarity Coefficient (DSC) ranging from 0.766 to 0.809 for GTV-T and from 0.623 to 0.698 for GTV-N (54, 55). The only study in which the CTV was autosegmented showed a good agreement with manual delineation, achieving a DSC of 0.826, and outperforming the results of the previously published convolutional neural network (CNN), visual geometry group-16 (VGG-16) (55). Notably, the use of a semiautomated method for GTV-T segmentation proved to be less time consuming and correlated with an increase in the intra- and interoperator agreement when compared with fully manual segmentation (52).

Among algorithms for OAR delineation, studies were heterogeneous in the choice of the target(s) of segmentation. The majority of studies (12/13) considered PG segmentation as a primary endpoint (56–68), with the PG being the only considered region of interest (ROI) in four of the selected works (63, 65–67). The segmentation performance assessed by the DSC for all OARs investigated in the included studies is provided in **Table 2**.

**Table 2:** Reported Dice Similarity Coefficient (DSC) in literature for different organs.

Organ	No. of studies (*N* = 14)	Reference papers	DSC (median, IQR range)
**PG**	13	56–67, 100	0.84 (0.83–0.86)
**Mandible**	9	56–61, 64, 67, 100	0.93 (0.90–0.94)
**Brainstem**	8	56–61, 67, 100	0.86 (0.84–0.89)
**Optic nerves**	7	56, 58–61, 64, 67	0.69 (0.67–0.71)
**Submandibular glands**	7	56, 58–61, 64, 67, 100	0.80 (0.76–0.81)
**Chiasm**	5	56, 59, 61, 64, 68	0.532 (0.412–0.581)
**Spinal cord**	4	57, 58, 60, 64	0.88 (0.77–0.96)
**Oral cavity**	3	57, 58, 100	0.90 (0.80- 0.91)
**Eyeballs**	2	57, 64	0.91
**Lenses**	2	57, 60	0.86
**Temporomandibular joint**	2	57, 64	0.85
**Cochleae**	2	58, 60	0.82^a^
**Pharyngeal constrictors**	2	58	0.57^b^
**Glottic region**	2	58, 100	0.57^c^
**Brain**	1	60	0.99^c^
**Lacrimal glands**	1	60	0.65^c^
**Orbits**	1	60	0.93^c^
**Spinal canal**	1	60	0.84^c^
**Lungs**	1	60	0.98
**Upper esophagus**	1	58	0.69
**Supraglottic larynx**	1	58	0.77
**Larynx**	1	57	0.87
**Mastoids**	1	57	0.82
**Whole pharynx**	1	64	0.69

a Vandewinckele et al. (57) achieved a DSC of 0.65 with the use of CNN and Nikolov et al. (59) a DSC of 0.982 by a 3D U-Net.

b The reported DSC was computed as an average of inferior, medial and superior.

c The average value of two (in some cases three) models was considered.

Overall, autosegmentation studies were mainly CT based (13/21); in decreasing order of frequency were MR (three of 21), CT + MR (two of 21), positron emission tomography (PET, two of 21), and CT + PET (one of 21). Sample size varied considerably, ranging from 5 to 486 (median: 46, IQR: 15–166).

A complete description of individual studies characteristics is provided in **Table 3**.

**Table 3:** Characteristics for machine-learning studies on autosegmentation.

Author, year of publication	Study population	HN subsite	Imaging modality	Textural and dosimetric parameters	ROI(s)	Tested ML algorithm(s)	Statistical findings and model performance
Brunenberg et al., 2020 (68)	58 pts	Mixed	CT	–	PGs, SMGs, thyroid, buccal mucosa, extended OC, pharynx constrictors, cricopharyngeal inlet, supraglottic area, MNDB, BS	Commercially available DL model; external validation	The best performance was reached for the MNDB (DSC 0.90; HD95 3.6 mm); the agreement was moderate for the aerodigestive tract with the exception of the OC. The largest variations were in the caudal and/or caudal directions (binned measurements).
Ma et al., 2019 (69)	90 pts	NPC	CT and MR	–	GTVs	CNNs	Both M-CNN and C-CNN showed better performance on MR than on CT. C-CNN outperformed M-CNN in both CTs (higher mean Sn, DSC, and ASSD, comparable mean PPV) and MR applications (higher mean PPV, DSC, and ASSD, comparable mean Sn)
Vandewinckele et al., 2019 (58)	9 pts	Mixed	CT	–	Cochlea, BS, upper esophagus, glottis area, MNDB, OC, PGs, inferior, medial and superior PCMs, SC, SMGs, supraglottic Lar	CNN	The longitudinal CNN is able to improve the segmentation results in terms of DSC compared with the DIR for 6/13 considered OARs. The longitudinal approach outperforms the cross-sectional one in terms of both DSC and ASSD for 6 different organs (BS, upper esophagus, OC, PGs, PCM medial, and SMGs)
Hänsch et al., 2018 (63)	254 pts, 254 R PGs, 253 L PGs	Mixed	CT	–	Ipsi- and contralateral PGs	DL U-net	The 3 ANNs showed comparable performance for training and internal validation sets (DSC ≈0.83). The 2-D ensemble and 3-D U-net showed satisfactory performance when externally validated (AUC and DSC: 0. 865 and 0.880, respectively; 2-D U-net omitted)
Mocnik et al., 2018 (62)	44 pts	Not specified	CT and MR	–	PGs	CNN	The multimodal CNN (CT + MR) compared favorably with the single modality CNN (CT only) in the 80.6% of cases. Overall, DSCs value were 78.8 and 76.5, respectively. Both multi- and single-modality CNNs showed satisfactory registration performance
Nikolov et al., 2018 (60)	486 pts, 838 CT scans for training, test and internal validation; 46 pts and 45 CT scans for external validation	Mixed	CT	–	Brain, BS, L and R cochlea, L and R LG, L and R Lens, L and R Lung, MNDB, L and R ON, L and R Orbit, L and R PGs, SC, L and R SMG	3D U-Net	The segmentation algorithm showed good generalizability across different datasets and has the potential of improving segmentation efficiency. For 19/21 performance metrics (surface and volumetric DSC) were comparable with experienced radiographers; less accuracy was demonstrated for brainstem and R-lens
Ren et al., 2018 (70)	48 pts	Not specified	CT	–	Chiasm, L and R ON	3D-CNNs	The proposed segmentation method outperformed the one developed by the MICCAI 2015 challenge winner for all the considered ROIs (DSC chiasm: 0.58 ± 0.17 *vs*. 0.38; DSC ONs 0.71 ± 0.08 *vs*. 0.68)
Tong et al., 2018 (61)	32 pts	Not specified	CT	–	L and R PGs, BS, Chiasm, L and R ONs, MNDB, L and R SMG	FCNN with and without SRM	Accuracy and robustness of the model were improved when incorporating shapes prior to SRM use for all considered ROIs. Segmentation results were satisfactory, ranging from DSC values of 0.583 for the chiasm to 0.937 for the MNDB. Average time for segmenting the whole structure set was 9.5 s
Zhu et al., 2018 (59)	271 CT scans	Not specified	CT	–	BS, Chiasma, MNDB, L and R ON, L and R PG, L and R SMG	Implemented 3D U-Net (AnatomyNet)	The AnatomyNet allowed for an average improvement in segmentation performance of 3.3% (DSC) as compared with previously published data of the MICCAI 2015 challenge. Segmentation time was 0.12 s for the whole structure set.
Doshi et al., 2017 (53)	10 pts/102 MR slices	Mixed	MR	–	GTVs	FCLSM	PLCSF showed a good performance *vs* the consensus manual outline (DSC: 0.79, RAD: 39.5%, MHD: 2.15, PCC: 0.89, *p* < 0.05) and outperformed 2 Ncut and MS clustering algorithms (the former being less accurate for small lesions and for low-contrast regions and more computationally demanding, the latter leading to more frequent over-segmentation)
Ibragimov et al., 2017 (64)	50 pts	Not specified	CT	–	SC, MNDB, PGs, SMGs, Lar, Phar, R and L EB, R and L ON, optic chiasm	CNN-MRF	Model performance was satisfactory for almost all considered OARs (DSC values as follows—spinal cord: 87 ± 3.2; mandible: 89.5 ± 3.6; PGs DSC: 77.3 ± 5.8; submandibular glands DSC: 71.4 ± 11.6; Lar DSC: 85.6 ± 4.2; phar DSC: 69.3 ± 6.3; eye globes DSC: 88.0 ± 3.2; optic ONs DSC: 62.2 ± 7.2; optic chiasm: 37.4 ± 13.4)
Liang et al., 2017 (55)	185 pts	NPC	CT	–	BS, R and L EB, R and L lens, Lar, R and L MNDB, OC, R and L MAS, SC, R and left PG, R and L T-M, R and L ON	CNNs (ODS-net)	ODS-net showed satisfactory Sn and Sp for most OARs (range: 0.997–1.000 and 0.983–0.999, respectively), with DSC >0.85 when compared with manually segmented contours. ODS-net outperformed a competing FCNN (*p* < 0.001 for all organs). Image delineation was faster in ODS than in FNC, as well, with average time of 30 *vs*. 52 s, respectively
Men et al., 2017 (55)	230 pts	NPC	CT	–	GTV-T, GTV-N, CTV	DDNN	DDNN generated accurate segmentations for GTV-T and CTV (ground truth: manual segmentation), with DSC of 0.809 and 0.826, respectively, Performance for GTV-N was less satisfactory (DSC: 0.623). DDNN outperformed a competing model (VGG-16) for all the analyzed segmentations
Stefano et al., 2017 (72)	4 phantom experiments+ 18 pts/40 lesions	Mixed	PET	–	GTVs	RW	Both the K-RW and the AW-RW compare favorably with previously developed methods in delineating complex-shaped lesions; accuracy on phantom studies was satisfactory
Wang et al., 2017 (56)	111 pts	Mixed	CT	–	Cochlea, BS, upper esophagus, glottis area, MNDB, OC, PGs, inferior, medial and superior PCMs, SC, SMGs, supraglottic Lar	3D U-Net	The model showed satisfactory performance for most of the 9 considered ROIs; when compared with other models, it ranked first in 5/9 cases (L and R PG, L and R ON, L SMG), and second in 4/9 cases
Beichel et al., 2016 (52)	59 pts/230 lesions	Mixed	PET	–	GTVs	Semiautomated segmentation (LOGISMOS)	Segmentation accuracy measured by the DSC was comparable for semiautomated and manual segmentation (DSC: 0.766 and 0.764, respectively)
Yang et al., 2014 (65)	15 pts/30 PGs/57 MRs	Mixed	MR	–	Ipsi- and contralateral PGs	SVM	Average DSC between automated and manual contours were 91.1% ± 1.6% for the L PG and 90.5% ± 2.4% for the R PG. Performance was slightly better for the L PG, also when assessed per the averaged maximum and average surface distance
Cheng G et al., 2013 (66)	5 pts, 10 PGs	NPC	MR	–	Ipsi- and controlateral PGs	SVM	Mean DSC between automated and physician’s PG contours was 0.853 (range: 0.818–0.891)
Qazi et al., 2011 (67)	25 pts	Not specified	CT	I	MNDB, BS, L and R PG, L and R SMG, L and R node level IB, L and R node levels II–IV	Atlas based segmentation	As compared with manual delineations by an expert, the automated segmentation framework showed high accuracy with DSC of 0.93 for the MNDB, 0.83 for the PGs,.83 for SMGs and 0,.74 for nodal levels
Chen et al., 2010 (54)	15 pts/15 neck nodal levels	Mixed	CT	–	II, III, and IV neck nodal levels	ASM	The ASM outperformed the atlas-based method (ground truth: manually segmented contours), with higher DSC (10.7%) and lower mean and median surface errors (−13.6% and −12.0%, respectively)
Yu et al., 2009 (73)	10 pts/10 GTV-T and 19 GTV-N	Mixed	PET and CT	I	GTVs	KNN	The feature-based classifier showed better performance than other delineation methods (e.g. standard uptake value of 2.5, 50% maximal intensity and signal/background ratio)

2D/3D, 2/3-dimensional; ANN, Artificial Neural Network; ASM, active shape model; ASSD, average symmetric surface distance; AW-RW, K-RW algorithm with adaptive probability threshold; BS, brainstem; CNN, convolutional neural network; C-CNN, combined CNN, CT, computed tomography; CTV, clinical target volume; D, dosimetric; DDNN, deep deconvolutional neural network; DIR, deformable image registration; DL, deep learning; DSC, Dice Similarity Coefficient; EB, eyeball; FCLSM, modified fuzzy c-means clustering integrated with the level set method; FCNN, fully convolutional neural network; GTV-N, nodal-gross tumor volume; GTV-T, tumor-gross tumor volume; HD, Hausdorff distance; I, imaging; KNN, k-nearest neighbors; K-RW, RW algorithm with K-means; L, left; Lar, larynx; LG, lacrimal gland; LOGISMOS, layered optimal graph image segmentation of multiple objects and surfaces; M-CNN, multimodality convolutional neural network; MHD, modified Hausdorff distance; MICCAI, Medical Image Computing and Computer Assisted Intervention; MNDB, mandible; MR, magnetic resonance; MRF, Markov random field; MAS, mastoid; MS, mean shift; Ncut, normalized cut; NPC, nasopharyngeal carcinoma; OAR, organ at risk; LG, lacrimal gland; OC, oral cavity; ODS-net, organs at risk detection and segmentation network; ON, optic nerve; p, p-value; PCC, Pearson correlation coefficient; PCM, pharyngeal constrictors muscles; PET, positron emission tomography; PG, parotid gland; Phar, pharynx; PLCSF, pharyngeal and laryngeal cancer segmentation framework; PPV, positive predictive value; pt, patient; R, right; RAD, relative area difference; ROI, region of interest; RW, Rescola Wagner; SC, spinal cord; s, second; SMG, submandibular gland; Sn, sensitivity; Sp, specificity; SRM, shape representation model; SVM, support vector machine; VGG-16, visual geometry group-16.

### Treatment Planning

Of the included studies, two focused on the identification of predictive factors for replanning (74, 75). Guidi et al. (74) used support vector machine (SVM) on a retrospectively collected cohort of 40 HNC patients and 1,200 megavoltage CTs to recognize those who could benefit from ART based on weekly anatomical and dosimetric divergences in CTV and OARs (namely, spinal cord, mandible, and PGs) during the course of treatment. Specifically, the authors could demonstrate that from the fourth week, 77% of patients underwent significant morphological and dosimetric changes, advocating the need for replanning. Of note, PGs were the most prone to modifications, with significant variations from the original plan occurring as early as from the third week of treatment. In the second study, Yu et al. (75) used radiomic features from contrast-enhanced T1-weighted and T2-weighted pre-RT MR images and Least Absolute Shrinkage and Selection Operator (LASSO) logistic regression to build models predicting the need of treatment replanning in a retrospective cohort of 70 patients with nasopharyngeal carcinoma (NPC). The combined T1–T2 model outperformed the ones based on either single MR sequence, with average areas under the curve (AUCs) in the training and testing sets of 0.984 (95% confidence interval (CI): 0.983–0.984) and 0.930 (95% CI: 0.928–0.933), respectively, and six radiomic features selected as significant.

A third study on ML for RT planning was published by Nguyen et al. (76) and focused on the use of a hierarchically densely connected U-net architecture (HD U-net) to predict three-dimensional dose distribution for the planning target volume and 22 OARs in a retrospectively retrieved population of 120 HNC patients. When compared with two variant net architectures (namely, Standard U-net and DenseNet), the proposed algorithm showed better performance in the prediction of the maximum and mean dose to the OARs, better dose homogeneity, conformity, and coverage on the test data. Additionally, the HD U-net requires fewer trainable parameters and a reduced computational time when compared with the Standard U-net and with the DenseNet, respectively.

Finally, Thummerer et al. (77) in their study compared synthetic CT images (sCTs) derived from cone-beam CTs (CBCTs) and MRs for HN patients in terms of both image quality and accuracy in proton dose calculation, considering planning CTs as the ground truth. Image quality was quantified through mean absolute error (MAE) and DSC. The sCTs from CBCTs provided higher image quality with an average MAE of 40 ± 4 HU and a DSC of 0.95, while for MR-based sCTs a MAE of 65 ± 4 HU and a DSC of 0.89 were observed. Overall, the study reports that CBCT- and MR-based sCTs have the potential to be reliably implemented into the ART workflow for proton therapy application, thus overcoming the need of performing multiple planning CTs.

### Oncological Outcome Prediction

Overall, 12 of the included studies considered oncological outcomes following curative-intent treatment as their target of prediction. In details, six studies (40, 42, 78–81) aimed at predicting OS, while five (40, 82–85) considered loco-regional control (LRC) and one (86) distant metastasis-free survival (DMFS). Only two works focused on more than one oncological outcomes (40, 87). Feature selection methods were applied in two cases (40, 42), both studies used radiomic features extracted from the GTV as input parameters for outcome prediction. Other than these works, four additional publications included texture analysis; overall, features were derived from CT images in two works (40, 86), from MR images in one (84) and from multiple diagnostic modalities in the remaining three cases (42, 82, 83).

A single disease subsite was considered by two studies, with Zdilar et al. (40) including only patients with oropharyngeal cancer (OPC), and Jiang et al. focusing on patients diagnosed with neoplasms of the nasopharynx. Conversely, Bryce et al. (79) and Parmar et al. (42) applied ML to mixed HNC populations; information on subsite distribution could be retrieved in only one case (79). Despite relevant heterogeneity in the choice of ML algorithms and populations, the best performing models in each study reached an AUC between 0.72 and 0.78; the best performance was reached by the only study using Artificial Neural Networks (ANNs) (79).

LRC was the target of prediction in four cases (40, 82–84); population size varied considerably, from the 32 NPC patients included in the study by Tran et al. (82) to the 529 patients diagnosed with OPC in the study published by Zdilar et al. (40). All studies considered the radiomic features extracted from the pretreatment GTV as input parameters for model construction. Three studies evaluated ML models through AUC values (40, 82, 83), with the best performing models being k-nearest neighbors and ANNs; Fujima et al. (84) assessed the performance of their nonlinear SVM models by sensibility, specificity, and positive and negative predictive values (for further details, please refer to **Table 4**).

**Table 4:** Characteristics for machine-learning studies on oncological outcome.

Authors, publication year	Sample study population	HN subsite	Clinical endpoint	Imaging modality	Textural and dosimetric parameters	ROI(s)	Tested ML algorithm(s)	Statistical findings and model performance
De Felice et al, 2020 (80)	273 pts	OPC	OS prediction in OPC pts treated with IMRT	None	–	None	Decision trees	The most relevant clinical variables identified were HPV status, nodal stage and early complete response to IMRT
Howard et al, 2020 (81)	33,527 pts	Mixed	OS prediction in HNC pts with intermediate risk factors treated with adjuvant CHT-RT or RT; identification of which pts may benefit from CHT-RT	None	–	None	DeepSurv, RSF, N-MLTR	Indication to treatment according to model recommendations was associated with a survival benefit; the best performance was achieved by DeepSurv, with an HR of 0.79 (95% CI, 0.72–0.85; *p* < 0.001). No survival benefit was observed for CHT in case pts were recommended for RT alone
Starke et al, 2020 (85)	291 pts	Mixed	LRC in locally-advanced HN SCC treated with primary CHT-RT	CT	–	GTVs	3D- and 2D-CNNs (from scratch, transfer learning and extraction of deep autoencoder features)	The best performance was achieved by an ensemble of 3D-CNNs (C-index = 0.31 on the external validation cohort); the model yielded a satisfactory performance in discriminating high- vs. low-risk LRC (*p* = 0.001)
Tseng et al, 2020 (87)	334 pts	OC	Risk stratification of locally-advanced OC pts treated with surgery	None	–	None	Elastic net penalized	The incorporation of genetic information to clinicopathologic data led to better model performance for the prediction of both CSS and LRC, as compared with models using clinicopathologic variables alone (mean C index, 0.689 vs. 0.673; *p* = 0.02 for CSS and 0.693 vs. 0.678; *p* = 0.004 for LRC). No such difference was noted for the prediction of DMFS
							Cox proportional hazards regression-based risk stratification model	
Fujima et al., 2019 (84)	36 pts	SNC	LC following superselective arterial CDDP infusion and concomitant RT	MR	I	GTVs (necrotic and cystic areas excluded)	Nonlinear SVM	Mean Sn: 1.0, Sp 0.82, PPV 0.86, NPV 1.0 (on validation data sets, 9-fold crossvalidation scheme used)
Tran et al., 2019 (82)	32 pts	NPC	RT response of metastatic nodes by ultrasound-derived radiomic markers	CT, MR, EUS	–	GTVs	LR, naive Bayes, and k-NN	There was a statistically significant difference in the pretreatment QUS-radiomic parameters between radiological complete responders vs. partial responders (*p* < 0.05). The best classification was achieved by k-NN with a single feature, SS-contrast (AUC = 0.866 [0.73; 1.01]); %Sn = 85.8; %Sp = 97.3; %Acc = 91.5)
Wu et al., 2019 (86)	140 pts	OPC	DMFS	CT	I	Baseline and mid-treatment GTV-T and GTV-N	RSF	Better performance on testing set was achieved by the model incorporating mid-treatment characteristics (C-index: 0.73, *p* = 0.008) vs. the model based on pretreatment CT features alone. The main features for DMFS prediction were: maximum distance among nodes, maximum distance between tumor and nodes (mid-treatment), and pretreatment tumor sphericity
Li et al., 2018 (83)	306 pts	NPC	Analyze the recurrence patterns in pts with NPC treated with IMRT	CT, MR and PET	I	GTVs	ANN, k-NN, and SVM	NPC-IFRs vs NPC-NPDs could be differentiated by 8 features (AUCs: 0.727–0.835). The classification models showed potential in prediction of NPC-IFR with higher accuracies (ANN: 0.812, KNN: 0.775, SVM: 0.732)
Zdilar et al., 2018 (40)	529 pts, >3,800 radiomic features	OPC	OS and RFS	CT	I	GTVs	Feature selectors: MRMR, Wilcoxon rank sum test, RF, RrliefF, RRF, IAMB, RSF, PCA	RF features selectors achieved the best performance for both OS prediction (AUC: 0.75, C-index: 0.76, calibration: 0.87) and. RFS (AUC: 0.71, C-index 0.68, calibration: 19.1). The ensemble model (clinical+ radiomic) yielded the best scores for AUC and C-index in all cases
							Predictive models: LR, CPH, RF, RSF, logistic elastic net, ensemble models	
Jiang et al., 2015 (78)	347 pts	NPC	OS prediction in pts with ab initio metastatic NPC (M1a vs. M1b)	None	–	None	SVM	The SVM classifier showed good performance at internal validation (AUC: 0.761, Sn 80.7%, Sp: 71.3%), while performance was less satisfactory when externally validated (AUC: 0.633)
Parmar et al., 2015 (42)	136 pts	Mixed	OS	CT and PET	–	GTVs	Feature selectors: RELF, FSCR, Gini, JMI, CIFE, DISR, MIM, CMIM, ICAP, TSCR, MRMR, MIFS, Wilcoxon	The three feature selection methods minimum redundancy maximum relevance (AUC = 0.69, stability = 0.66), mutual information feature selection (AUC = 0.66, stability = 0.69) and conditional infomax feature extraction (AUC = 0.68, stability = 0.7) had high prognostic performance and stability. The highest prognostic performance was achieved by GLM (median AUC ± SD: 0.72 ± 0.08) and PLSR (median AUC ± sd: 0.73 ± 0.07), whereas BAG (AUC = 0.55 ± 0.06), DT (AUC: 0.56 ± 0.05), and BST (AUC = 0.56 ± 0.07) showed lower AUC values. RF (RSD = 7.36%) and BAG (9.27%) were more stable classification methods, whereas PLSR (RSD = 12.75%) and SVM (RSD = 12.69%) showed lower stability
							Predictive models: NN, Decision tree, Boosting, Bayesian Bagging, RF, Multi adaptive regression splines (MARS), SVM, k-NN, GLM, partial least squares, and principal component regression	
Bryce et al., 1998 (79)	95 pts	Mixed	Survival prediction in pts with advanced HN SCC treated with RT ± chemotherapy	None	–	None	LR, ANN	ANNs compared favorably with LR models at survival prediction, with a AUC of 0.78 ± 0.05 for the best ANN and of 0.67 ± 0.05 for the best LR model. The best ANN outperformed the modified AJCC TNM 4th edition in survival prediction, as well. Incorporated clinical parameters for the ANN were: tumor size, tumor resectability, nodal stage, tumor stage, and baseline hemoglobin levels

ANN, Artificial Neural Network; AUC, area under the curve; CDDP, cisplatin; CHT, chemotherapy; CIFE, conditional infomax feature extraction; CMIM, conditional mutual information maximization; CNN, convolutional neural network; CSS, cancer-specific survival; CT, computed tomography; D, dosimetric; DISR, double input symmetric relevance; DMFS, distant metastasis free survival; GTV, gross tumor volume; HN, head and neck; HR, Hazard ratio; I, imaging ICAP, interaction capping; IMRT, intensity modulated RT; JMI, joint mutual information; k-NN, k-nearest neighbor; LC, local control; LR, logistic regression; LRC, loco-regional control; MARS, multiadaptive regression splines; MIFS, mutual information feature selection; MIM, mutual information maximization; MR, magnetic resonance; MRMR, minimum redundancy maximum relevance; NN, neural network; N-MLTR, neural network multitask logistic regression; NPC, nasopharyngeal cancer; OC, oral cavity cancer; OPC, oropharyngeal cancer; OS, overall survival; PET, positron emission tomography; PLSR, partial least square regression; RF, random forest; RFS, relapse-free survival; RSD, relative standard deviation; RSF, random survival forest; RT, radiotherapy; SCC, squamous cell carcinoma; SN, sinonasal cancer; SVM, support vector machine; TSCR, t-test score.

Lastly, the prediction of DMFS was the objective of one study (86). Wu et al. proved that the incorporation of pre- and mid-treatment radiomic features extracted from both the primary and nodal GTVs improved the performance of random survival forest models trained and validated on a cohort of 140 locally advanced OPC patients (86).

### Toxicity Outcome Prediction

A total of 11 studies focused on RT-induced toxicities; in each publication algorithms were developed for addressing the prediction task on a single outcome (i.e., xerostomia, dysphagia).

Four studies (), predominantly encompassing multiple HN subsites, focused on xerostomia prediction; all but one included dosimetric parameters in the data set (88). The PGs were the only considered ROI except for the work by Guo et al. (89), where the submandibular glands were included. Despite the common clinical focus, different endpoints for the task of xerostomia prediction were considered. Acute xerostomia was the focus of one study, which aimed to predict parotid shrinkage (88), late xerostomia was investigated in one publication (45), while the development of xerostomia at any time following RT was considered by Soares et al. (90). Gabrys et al. built distinct algorithms for the prediction of early, late, and long-term xerostomia; longitudinal models were developed as well (91). Notably, ML-based classifiers outperformed classic Normal Tissue Complication Probability (NTCP) models based on the sole mean dose to the parotids, thus underlying the need of incorporating multiple parameters for accurate outcome prediction (i.e., gland volume and dose gradients in the right-left and anterior-posterior direction for long-term xerostomia). Overall, sample size was comparable across studies focusing on xerostomia prediction (138–153), except for the one by Pota et al., which analyzed 21 patients (88).

The remaining studies presented different toxicity outcomes (namely, acute dysphagia, weight loss at 3 months following the end of RT, osteoradionecrosis, sensorineural loss, and brain injury) (46, 92–95). A full list of the developed algorithms and statistical findings for all studies included in this subsection is provided in **Table 5**.

**Table 5:** Characteristics for machine learning studies on toxicity outcome.

Author, year of publication	Study population	HN subsite(s)	Clinical endpoint	Imaging modality	Textural and dosimetric parameters	ROI(s)	Tested ML algorithm(s)	Statistical findings and model performance
Humbert-Vidan et al, 2021 (95)	96 pts (of these, 50% controls)	Mixed	Prediction of osteoradionecrosis of the mandible	CT	D	Mandible	LR, SVM, RF, AdaBoost, ANN	No statistically significant difference was found among the models in terms of either accuracy, TPR, TNR, PPV, NPV).
Zhang et al, 2020 (94)	242 pts	NPC	Early radiation-induced brain (temporal lobes) injury prediction	MRI	I	Temporal lobes	RF (3 models)	The incorporation of textural features yielded to better model performance; features derived from T2-w images achieved higher performance than those extracted from T1-w images. In the testing cohort, models 1, 2, and 3, yielded AUCs of 0.830 (95% CI: 0.823–0.837), 0.773 (95% CI: 0.763–0.782), and 0.716 (95% CI: 0.699–0.733), respectively.
Guo et al., 2019 (45)	146 pts	PGs	Correlation between voxel dose and xerostomia recovery 18 months after RT	None	D	PGs, SMGs	LR with ridge regularization	The AUC scores for the ridge logistic regression model evaluated by 10-fold crossvalidation for recovery and injury prediction were 0.68 ± 0.07 and 0.74 ± 0.03, respectively.
Leng et al., 2019 (93)	77 pts, 67 healthy controls	NPC	Identification of biomarkers of WM injury *via* MR DTI, TBSS, and ML	MR	–	116 brain regions (90 for the brain lobes and 26 for the cerebellum) per the AAL method	SVM	WM regions and WM connections were involved in RBI. The SVM classifier showed satisfactory performances (GR, Sn, Sp) for both FA and WM connections in discriminating patients and controls at all-time points (0–6, 6–12, >12 months)
Abdollahi et al., 2018 (92)	47 pts, 94 cochleas, 490 radiomic features	Mixed subsites	Sensorineural hearing loss prediction following chemoradiotherapy	CT	I, D	Cochlea	Decision stump, Hoeffding, C4.5, Bayesian network, naive, adaptive boosting, bootstrap aggregating, Classification *via* regression, logistic regression, linear logistic	Predictive power was >70% for all models, with Decision stump and Hoeffding being the best-performing models. Incorporation of the gEUD improved both precision and AUC of all models, while accuracy was not affected
Dean et al., 2018 (46)	173 pts + 90 pts for external validation	Mixed subsites	Peak grade of acute dysphagia prediction (severe = CTCAE 3.0 grade ≥3 vs. nonsevere = CTCAE 3.0 grade <3)	None	D	Pharyngeal mucosa	PLR, SVC, RFC (each trained and validated on standard dose-volume metrics and spatial dose-metrics)	PLR was not outperformed by any of the more complex models, on both internal and external validation (AUC: 0.76 and 0.82 for the standard-dose model and AUC: 0.75 and 0.73 for the spatial model, respectively). Calibration was superior for the RFC model. Dosimetric parameters (DVH, DLH and DCH) were relevant for accurate toxicity prediction: the volume of pharyngeal mucosa receiving ≥1 Gy should be minimized
Gabrys et al., 2018 (91)	153 pts, 24 selected radiomic features	Mixed subsites	Evaluation of xerostomia risk prediction with integrated ML models (clinical, dosimetric, and radiomic features) vs. NTCP models based on mean RT dose to the PGs	CT	I, D	Ipsi- and contralateral PGs	LR-L1, LR-L2, LR-EN, kNN, SVM, ET, GTB	SVMs were the top performing classifiers in time-specific xerostomia prediction (early, late, long term). In the longitudinal approach, the best models were GTB, ET and SVM. LR models were the best in feature selection, although selecting features did not provide any improvement in predictive performance. The NTCP mean dose-based models failed to predict xerostomia (AUC <0.60)
Cheng Z et al., 2017 (96)	391 pts	Mixed subsites	Prediction of WL ≥5 kg at 3 months post-RT	None	D	Pharyngeal constrictors, cricopharyngeus, masticator, temporalis, pterygoids, oral cavity, oral mucosa, soft palate, larynx, parotid gland, submandibular glands	CART algorithms	CART model encompassing toxicity and QoL data performed better than the one including baseline characteristics and dosimetric data (AUC: 0.82 vs. 0.77, Sn: 0.98 vs. 0.77, Sp 0.59 vs. 0.67, PPV 0.46 vs. 0.43, NPV: 0.99 vs. 0.90, respectively)
Soares et al., 2017 (90)	138 pts	Mixed subsites	Predicting xerostomia after RT	None	D	PGs	RF, stochastic boosting, SVM, NN, model-based clustering and LR	RF yielded the best model performance (AUC: 0.73); the incorporation of clinical (gender, age, baseline xerostomia) and dosimetric parameters (PG Dmean) outperformed all other RF combinations
Pota et al., 2015 (88)	21 pts, 42 parotids	NPC	Parotid gland shrinkage prediction	CT	I	Ipsi- and controlateral PGs	LFA, LDA, LR, 0-R method	In some cases, with only one predictor, the LR method presents the highest accuracy but low specificity, while in other cases with only one variable the performances of LDA, LR, and LFA are comparable. If more than one variable is used, the LFA classifier is the best in almost all the cases (best accuracy and sensitivity), while specificity is comparable with that of other classifiers. Adding a variable to a model hardly worsens the performances of both LDA and LR, while LFA models tolerate the noise

ANN, Artificial NN; AUC, area under the curve; CART, classification and regression tree; CT, computed tomography; CTCAE, common terminology criteria for adverse event; D, dosimetric; DCH, dose coverage histogram; DLH, dose lymphocyte histogram; Dmean, mean (RT) dose; DTI, diffusion tensor imaging; DVH, dose volume histogram; ET, extra-trees; gEUD, generalized equivalent uniform dose; GTB, gradient tree boosting; I, imaging; k-NN, k-nearest neighbor; LDA, linear discriminant analysis; LFA, logical framework approach; LR, logistic regression; ML, machine learning; MR, magnetic resonance; NN, neural network; NPC, nasopharyngeal cancer; NPV, negative predictive value; NTCP, normal tissue complication probability; OPC, oropharyngeal cancer; PG, parotid gland; PLR, penalized LR; PPV, positive predictive value; QoL, quality of life; RFC, random forest classification; SMG, submandibular gland; Sn, sensitivity; Sp, specificity; SVM, support vector machine; TNR, true-negative rate; TPR, true-positive rate; T1/T2-w, T1/T2-weighted; TBSS, tract-based spatial statistics; WL, weight loss; WM, white matter.

### Checklist Scores

**Figure 4:**
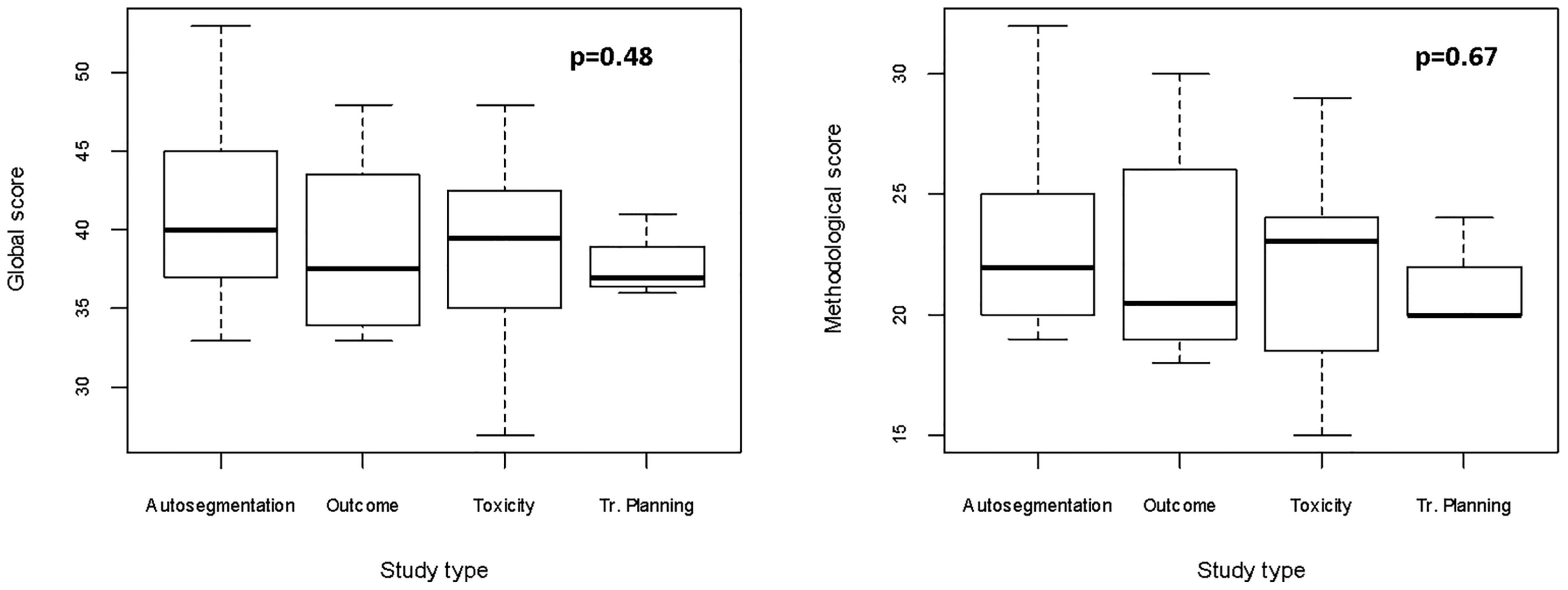
Boxplots for global and methodological scores (modified Luo classification) for the studies included in the analysis, categorized according to the task of the proposed algorithm(s). Tr, treatment.

Considering a maximum achievable score of 58 in the adapted Luo rating system for ML applications in biomedical research, median score of the included studies was 39 (IQR: 36–44), with minimum and maximum values being 27 and 53, respectively. When analyzing the *Methods* items only, median rank was 22 (IQR: 20–25), with the worst and best scores being 15 and 32, respectively. As it can be noted in **Figure 4**, the groups achieved comparable scores and no statistically significant difference was noted in studies global and methodological ranking (*p* = 0.48 and 0.67, respectively; FDR-corrected *p* = 0.62 and 0.67, respectively). Yet, studies dedicated to outcome modeling and treatment planning achieved numerically lower scores in both the global and methodological assessment.

**Figure 5:**
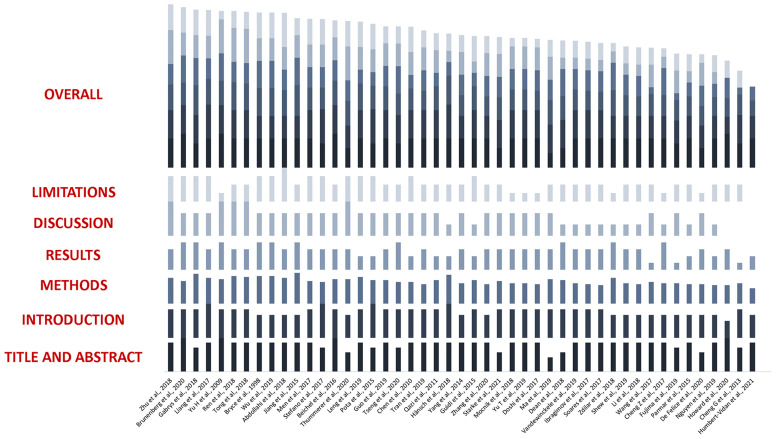
Boxplots for global and methodological scores (modified Luo classification) for the studies included in the analysis, categorized according to imaging data used as input parameters (texture analysis vs. no texture analysis).

The scores for studies implementing imaging data (*n* = 37) categorized according to the use of texture analysis vs. other imaging-derived metrics or deep learning (*n* = 10 and 27, respectively) were evaluated. Since the analysis of quantitative extracted features usually requires an intensive work of statistical preprocessing, frequently lacking in deep learning studies, we tested the hypothesis that studies extracting features are associated with higher methodological scores. Even though no significant difference was found, a trend favoring texture analysis publications was noted especially for methodological study quality (*p* = 0.45 [FDR-corrected *p* = 0.67] vs. *p* = 0.62 [FDR-corrected *p* = 0.62] when the global score was considered, as shown in **Figure 5**).

**Figure 6:**
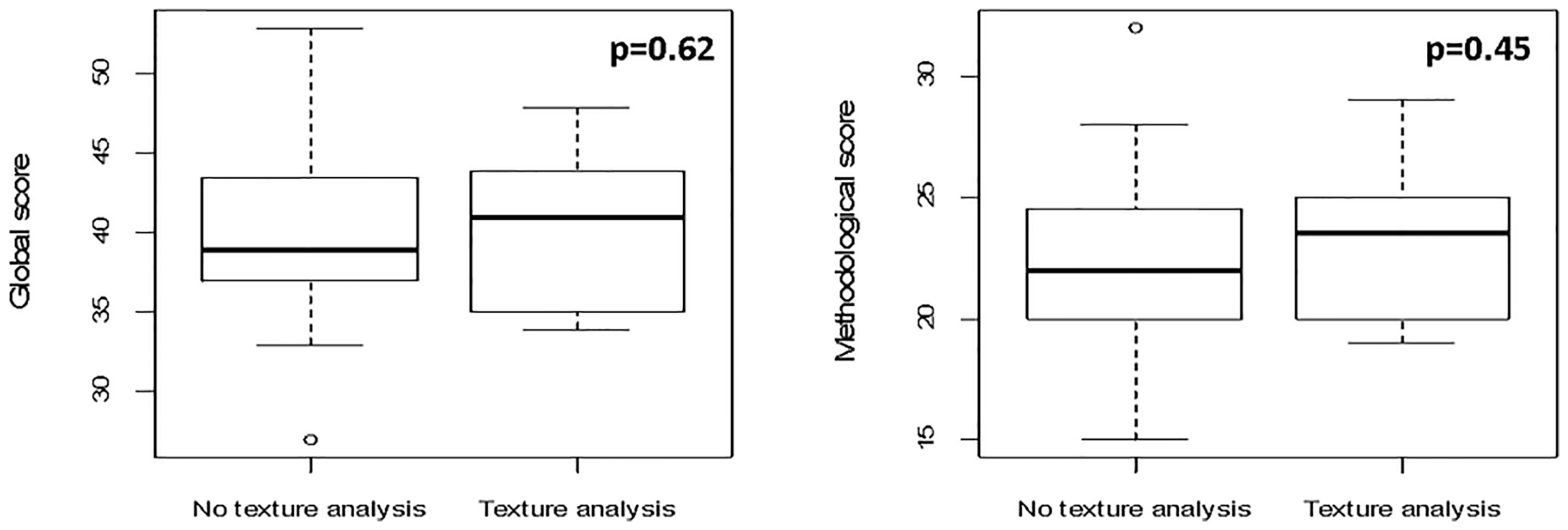
Boxplots representing global and methodological scores (modified Luo classification) for the studies included in the analysis, categorized per the presence of texture analysis.

The complete evaluation of each study is provided in **Figure 6**.

## Discussion

Results from our systematic review show a wide range of possible applications of ML in the field of HN Radiation Oncology, although this area of research is relatively young, with the majority of studies having been published in the last 3 years. The implementation of quantitative imaging features and the use of a longitudinally collected data as input parameters are both promising in refining model performance and open doors to further investigations.

The present analysis indicates a prevalence of algorithms dedicated to autocontouring, which mirrors the still unmet need for computationally affordable and user-friendly tools for clinical practice implementation. Even if only some authors have attempted to provide a full set of ROIs (56–61, 64, 67, 68), they could demonstrate a general improvement over existing models, with average times for task completion ranging between 0.12 and 30 s. However, the segmentation of small and/or low-contrasted areas, which are common in HN anatomy (e.g., optic chiasm, lenses, brainstem) remains challenging, and more efforts are warranted to equal, or at least to approximate, the performance of semiautomated or fully manual segmentation.

Currently available works on ML for treatment planning are scarce and show significant heterogeneity both in the choice of algorithms and in the characteristics of patients’ populations. Nevertheless, results are promising, as they pave the way to the possibility of effectively reconstructing three-dimensional dose distribution of integrating MR in ART and of predicting the need for replanning based on geometrical and dosimetric modifications during treatment. It is straightforward to understand how the fulfillment of these objective may be relevant in everyday clinical practice, especially in the era of image-guided IMRT for HNC (25). Additionally, reliable ML-based predicting tools may be beneficial also for proton treatment planning, as dose deposition is heavily influenced by patient’s set-up and anatomical variations of both target volumes and OARs (77, 97, 98).

Intriguing findings were reported for outcome prediction, as well. Considering oncological outcomes, supervised and unsupervised models were used with an overall satisfactory performance in small- to medium-sized datasets. Notably, the use of combined models incorporating radiomics (40) and longitudinal characteristics (86) yielded the best results. Moreover, neural networks outperformed competing algorithms in the prediction of recurrence patterns in NPC and survival in a population of locally advanced HNCs, respectively (79, 83). Conversely, only two studies incorporated ANNs for the prediction of RT-related toxicities (90, 95), and a prevalence of binary classifiers using labelled data was noticed, as expected. Gabrys et al. (91) were the only ones who compared ML univariate and multivariate logistic regression models to classical NTCP models based on the mean dose to the PGs. In their study, the authors could demonstrate that clinical characteristics and organ- and dose-shape features can improve xerostomia prediction, thus emphasizing the need of multidimensional input parameters to model complex outcomes.

Only one study focused on the use of ML for the analysis of organizational features of RT. In detail, Shew et al. (99) used a supervised classifier to discriminate risk factors correlating with delays in adjuvant treatment delivery. Despite several methodological limitations, the work is based on a large cohort from the National Cancer Database (NCDB), and includes a total of 76,573 patients. Another worth of this study relies in the use of ML for optimizing treatment scheduling: while prediction accuracy needs improving, the proposed model still provides a valuable example on how ML could be used in Radiation Oncology departments to facilitate executional tasks and, ultimately, to improve the quality of care.

Despite desirable, it is not currently possible to perform a reliable comparison among models, even for algorithms designed for the same task (i.e., autosegmentation). Not only was the choice of algorithms, features and variables widely heterogeneous, but most studies considered small- to medium-sized datasets and mixed disease subsites. In particular, sample size could strongly affect the quality of ML models as the training sets size is widely recognized as one of the main issues in pattern recognition studies. In fact, as the number of considered features increases, larger training sets become mandatory to avoid the so-called curse of dimensionality (100). To partially overcome this issue, we have performed a qualitative comparison based on a modified version of a reporting guideline validated by Luo et al. (23), which was previously introduced by Jethanandani et al. (12) in their systematic review on MR-based radiomic studies in HNCs. As pointed out by the authors, the checklist is not without limitations, including difficult and/or subjective interpretability of some items, as noted by our group as well.

Considering these pitfalls, and the fact that the checklist was not designed to provide a quantitative assessment, relevant findings still emerged. Firstly, studies aiming at toxicity prediction resulted to have the highest quality in both global and methodological scores as compared with those classified in the other categories. Secondly, works incorporating quantitative image features as input parameters had better median methodological scores, which could be at least partially explained by adequate reporting on the preprocessing on imaging data. Finally, works having a nonclinician as first author achieved a higher ranking, with a strong statistical significance. This finding could derive from the scarcity of dedicated educational training on ML and statistics in most medical schools and residency programs.

The DSC was the performance evaluation metric used in all works dedicated to autosegmentation, while the AUC was implemented in one study only (63). Considering the remaining publications, the AUC was the metric of choice in 17/27 (63%) cases. Despite its popularity for model assessment, limitations of the AUC have been extensively discussed (101). While a dissertation on the matter is beyond the scope of this work, those approaching ML should consider that AUC weights false positive and false negative predictions equally, which can be extremely relevant in the clinical setting (i.e., when the aim is to predict if a patient will develop mild vs. severe xerostomia).

Admittedly, our work presents some limitations. As for all systematic reviews, eligible publications of the last months may be missing, albeit the search was repeated regularly while the manuscript was being written. Moreover, despite our attempt to perform a comprehensive search, the lack of a common ontology in ML may have led to the exclusion of some works: to overcome this potential bias, cross-references from the included works were screened for eligibility. To conclude, we provided the full search strategy for future reference, as we are aware that several additional works will be published in the upcoming months, given the fast-growing nature of this field.

Acknowledging these issues, we do believe that, other than being a full overview of existing literature, the value of our work is to provide a systematic quality assessment of published works, which could be informative for both general and advanced readers. Large-scale datasets, common ontology, study design, and performance reporting will most probably be needed to concretely implement ML in clinical practice, and discussion on this regard is both expected and encouraged. To this aim, the inclusion of dedicated AI courses in the educational track of future ROs would arguably foster the quality of scientific outputs in the field.

Finally, ML-based modeling for HNC is a promising and rapidly expanding field, even though more solidly constructed and validated algorithms are warranted to overcome the boundaries of speculative investigation and to open doors to better tailored Radiation Oncology for this subset of patients. Overall, if not safe yet, ML is most probably a bet worth making.

## Data Availability Statement

The individual scores assigned to all the studies included in the manuscript are available upon request to the corresponding author.

## Author Contributions

SV, MP, MZ, FB, RS, GM, and BJF were responsible for conception and design of the study and wrote the first draft of the manuscript. SV, MP, MZ, and FB were responsible for data acquisition and wrote sections of the manuscript. LJI, DA, SG, AS, ML, and RO wrote sections of the manuscript. All authors contributed to manuscript revision and read and approved the submitted version.

## Funding

The study was fully funded by the University of Milan with APC funds. The Institution of some authors (IEO) was partially supported by the Italian Ministry of Health with Ricerca Corrente and 5 × 1,000 funds. SV was supported by the Department of Oncology and Hemato-Oncology (DIPO) of the University of Milan with “Progetto di Eccellenza”. MZ received a research grant from the European Institute of Oncology-Cardiologic Center Monzino Foundation (FIEO-CCM), with a project entitled “Proton therapy vs. photon-based IMRT for parotid gland tumors: a model based approach with Normal Tissue Complication Probability (NTCP)” outside the current study. SV, FB, and LJI are PhD students within the European School of Molecular Medicine (SEMM), Milan. The sponsors did not play any role in the study design, collection, analysis, and interpretation of data, nor in the writing of the manuscript, nor in the decision to submit the manuscript for publication.

## Conflict of Interest

The authors declare that the research was conducted in the absence of any commercial or financial relationships that could be construed as a potential conflict of interest.

## Publisher’s Note

All claims expressed in this article are solely those of the authors and do not necessarily represent those of their affiliated organizations, or those of the publisher, the editors and the reviewers. Any product that may be evaluated in this article, or claim that may be made by its manufacturer, is not guaranteed or endorsed by the publisher.

## Abbreviations

AI, artificial intelligence; ANN, Artificial Neural Network; ART, adaptive RT; AUC, area under the curve; CBCT, cone-beam CT; CI, confidence interval; CNN, Convolutional Neural Network; CT, computed tomography; CTCAE, common terminology criteria of adverse events; CTV, clinical target volume; DMFS, distant metastasis-free survival; DSC, Dice Similarity Coefficient; FDR, false-discovery rate; GTV, gross tumor volume; GTV-N, GTV-nodal; GTV-T, GTV-tumor; HD U-net, Hierarchically Densely connected U-net; HN, head and neck; HNC, HN cancer; IMRT, intensity-modulated RT; IQR, interquartile range; LASSO, Least Absolute Shrinkage and Selection Operator; LRC, loco-regional control; MAE, mean absolute error; ML, machine learning; MR, magnetic resonance; NCDB, National Cancer Database; NPC, nasopharyngeal carcinoma; NTCP, normal tissue complication probability; OAR, organ at risk; OPC, oropharyngeal cancer; OS, overall survival; PET, positron emission tomography; PG, parotid gland; PRISMA, Preferred Reporting Items for Systematic Reviews and Meta-Analysis; RO, radiation oncologist; ROI, region of interest; RT, radiotherapy; sCT, synthetic CT; SVM, support vector machine; VGG-16, Visual Geometry Group-16.
