# Supplementary material for: Machine Learning for Head and Neck Cancer: A Safe Bet?—A Clinically Oriented Systematic Review for the Radiation Oncologist
Source: Front Oncol. 2021 Nov 18;11:772663. doi: 10.3389/fonc.2021.772663 (PMC8637856; doi:10.3389/fonc.2021.772663)
Supplement: Supplementary file 1 [file DataSheet_1.doc]

**PUBMED**

(((((("Artificial Intelligence"[Mesh] OR "Logistic Models"[Mesh]) OR ("artificial neural network"[All Fields] OR "artificial neural networks"[All Fields] OR "computer neural network"[All Fields] OR "computer neural networks"[All Fields] OR "artificial intelligence"[All Fields] OR "deep learning"[All Fields] OR "machine learning"[All Fields] OR "support vector machine"[All Fields] OR "support vector machines"[All Fields] OR (support[All Fields] AND ("genetic vectors"[MeSH Terms] OR ("genetic"[All Fields] AND "vectors"[All Fields]) OR "genetic vectors"[All Fields] OR "vector"[All Fields] OR "disease vectors"[MeSH Terms] OR ("disease"[All Fields] AND "vectors"[All Fields]) OR "disease vectors"[All Fields]) AND machinery[All Fields]) OR "random forest"[All Fields] OR "naive bayes"[All Fields] OR "bayes classification"[All Fields] OR "decision tree"[All Fields] OR "decision trees"[All Fields])) OR ("logistic regression"[All Fields] OR "logistic regressions"[All Fields] OR (logistically[All Fields] AND regressed[All Fields]) OR "logistic model"[All Fields] OR "logistic models"[All Fields] OR "predictive model"[All Fields] OR "predictive models"[All Fields])) OR "Logistic Models"[Mesh]) OR ("logit model"[All Fields] OR "logit models"[All Fields])) AND ("head and neck neoplasms"[MeSH Terms] OR ("head"[All Fields] AND "neck"[All Fields] AND "neoplasms"[All Fields]) OR "head and neck neoplasms"[All Fields] OR ("head"[All Fields] AND "neck"[All Fields] AND "cancer"[All Fields]) OR "head and neck cancer"[All Fields])) AND ("radiotherapy"[Subheading] OR "radiotherapy"[All Fields] OR "radiotherapy"[MeSH Terms]) AND "adult"[MeSH Terms]

**EMBASE**

(('artificial intelligence' OR 'logistic model' OR 'artificial neural network' OR 'deep learning' OR 'machine learning' OR 'support vector machine' OR 'random forest' OR 'bayesian learning' OR 'decision tree' OR 'predictive model') AND 'oropharynx tumor' OR 'nasopharynx tumor' OR 'larynx tumor' OR 'hypopharynx tumor' OR 'mouth tumor') AND radiotherapy #1 AND [adult]/lim

**SCOPUS**

ALL ( "artificial intelligence"  OR  "logistic model*"  OR  "artificial neural network*"  OR  "deep learning"  OR  "machine learning"  OR  "support vector machine*"  OR  "random forest*"  OR  "bayes classification"  OR  "predictive model*"  OR  "decision tree" )  AND  ALL ( "head and neck cancer" )  AND  ( radiotherapy )  AND NOT  INDEX ( medline )
